# Supplementary material for: A Scalable Heat Pump Film with Zero Energy Consumption
Source: Polymers (Basel). 2022 Dec 29;15(1):159. doi: 10.3390/polym15010159 (PMC9823491; doi:10.3390/polym15010159)
Supplement: Supplementary file 1 [file polymers-15-00159-s001.zip › polymers-2079596-supplementary.pdf]

Supplemental Information

A Scalable Heat Pump Film with Zero Energy Consumption

Zhenghua Meng[1,2], Boyu Cao[3,4], Wei Guo[1,2], Yetao Zhong[3], Bin Li[3], Changhao Chen[3], Hengren Hu[3], Shigang Wu[5], Zhilin Xia[3] *

[1] Hubei Key Laboratory of Advanced Technology for Automotive Components, Wuhan University of Technology, Wuhan, Hubei 430070, PR China. [2] School of Automotive Engineering, Wuhan University of Technology, Wuhan, Hubei 430070, PR China.

[3] State Key Laboratory of Silicate Materials for Architectures, Wuhan University of Technology, Wuhan, Hubei 430070, PR China.

[4] School of Materials Science and Engineering, Wuhan University of Technology, Wuhan, Hubei 430070, PR China.

[5] School of Materials Science and Engineering, Shandong University of Technology, Zibo, Shandong 255049, PR China.

Corresponding author's email address:
xiazhilin@whut.edu.cn (Zhilin Xia)

Table of Contents

Supplementary Information 1: Heat Transfer Model for HPF
Supplementary Information 2: Conceptual model: Material's Infrared radiation distributes exponentially
Supplementary Information 3: Model calculation: The effect of the sun's absorption depth on the cooling performance of the radiator
Supplementary Information 4: Transmittance of PE, PP and POE film (all 25μm thickness) over MIR wavelength
Supplementary Information 5: Evaluation of infrared transmission properties of different particles
Supplementary Information 6: Effect of particle size of ZnO on the normalized scattering cross section
Supplementary Information 7: Particle choose
Supplementary Information 8: Various raw material price
Supplementary Information 9: Outdoor test experiment results of bubble films with different layers
Supplementary Information 10: Outdoor test experiment results of ZnO layer with different thickness
Supplementary Information 11: Measurement of transmittance of HPF
Supplementary Information 12: Comparison between different testing devices
Supplementary Information 13: Standard Deviation (S) of all-day outdoor test results
Supplementary Information 14: Test of insulation effect of HPF
Supplementary Information 15: Outdoor windshield experiments
Supplementary Information 16: Outdoor cooling experiments on HPF-covered building materials
Supplementary Information 17: The cooling performance of the material at different emitted and absorbed solar powers
Supplementary Information 18: High convection environment tests
Supplementary Information 19: Model calculation: Effects of surface convection heat transfer coefficients and number of bubble film layers
Supplementary Information 20: Bending tests: HPF vs. PEA

Supplementary Information 1: Heat Transfer Model for HPF

In the HPF optimization stage, in order to analyze the effect of solar reflection, thermal radiation intensity and thermal insulation capacity on cooling, we established the following model using the finite element analysis method. Considering all heat change processes distributed along volume of objects, for homogeneous materials, the distribution of radiation along the thickness direction satisfies:

$$P_r = A\int_0^x e^{-\mu x}\,dx \tag{1}$$

$$P_{r\,x} = A\,e^{-\mu x} \tag{2}$$

Where P_r is the material radiation power (W/m^2), $P_{r\,x}$ is the power distribution of the material along the x-thickness direction (W/m^3), A and μ are constants that determine the material radiation power magnitude and distribution. Using PDMS as the cooled object, the total power of PDMS was set to 130 W (exponentially distributed along 200 μm). The total power of ZnO was 70 W (exponentially distributed along 2.4 mm). The total power of bubble film was 80 W (exponentially distributed along 63 mm), where the bubble film is seen as a homogeneous mixture of air and PE.

The net cooling power (P_{net}) of a radiative cooler can be calculated by the equation below:

$$Q_{net} = Q_r - Q_a - Q_{nonrad} - Q_{sun} \tag{3}$$

Q_{sun} is the solar power absorbed by the sample (including HPF and PDMS). $Q_{sun} = 0$ (at night), $Q_{sun} = P_{sun} \cdot \varepsilon_1$ (daytime), where $P_{sun} = 800\mathrm{W}/m^2$, ε_1 is the solar absorption coefficient. Q_{sun} is given in the form of an exponential distribution.

Q_{nonrad} is nonradiative heating power obtained by the sample from the surrounding media.

$$Q_{nonrad} = h(T_{amb} - T_r) \tag{4}$$

where h is the non-radiant heat transfer coefficient ($h = \frac{1}{R_1+R_2+\frac{1}{Q_{conv}}}$). Due to the thin ZnO film, for HPF only the thermal resistance of the bubble film is considered ($R_1 = n \cdot \frac{2e-3}{0.04}$). n is the number of bubble film layers. A layer of bubble film is 2e-3m thick and the thermal conductivity is 0.04 W/m/K. The HPF and the radiator are not closely connected, and there is an air layer of 500μm in between (λ_{air}= 0.023W/m/K, $R_2 = \frac{500e-6}{0.023}$). Q_{conv} is the surface heat transfer coefficient.

Q_a is the incident atmospheric radiation absorbed by the sample. Atmospheric window transparency is set to 0.8.

$$Q_a = 2\Pi\int_0^{\frac{\Pi}{2}} sin\theta\; cos\theta\; d\theta \int_0^{\infty} B(T_a,\lambda)\; e_r(\lambda,\theta)\; e_a(\lambda,\theta) d\lambda. \tag{5}$$

Q_r is the radiative power emitted by the sample.

$$Q_r = P_{r\,ZnO} + \varepsilon_2 P_{r\,bubble\,film} + \varepsilon_2\varepsilon_3^n P_{r\,PDMS} \tag{6}$$

Considering the IR masking of the upper layer to the lower material, ε_2 is the IR transmittance of the ZnO film and ε_3 is the IR transmittance of the bubble film (0.8).

According to Equation 3, if in the initial state ($T_{amb} = T_r$, $Q_{net} > 0$), the sample can achieve cooling. When $Q_{net} = 0$, the temperature difference ($T_{amb} - T_r$) is

expected to reach a steady state, which means that no additional power is available to cool the heat sink further. Therefore, the temperature difference $(T_{amb} - T_r)$ can be used to quantitatively evaluate the performance of the selective radiator.

Supplementary Information 2: Conceptual model: Material's Infrared radiation distributes exponentially

The top layer of ZnO reflects the sunlight, and the temperature increases slightly in the thinner depth. The bottom bubble film layer plays the role of radiative cooling together with the object. In contrast, the radiating layer and the reflecting layer of the common radiator are integrated together. If their thickness is the same as our sample, the common radiative cooler has a high solar reflection depth. The cooling comparison is shown in the Figure S1.

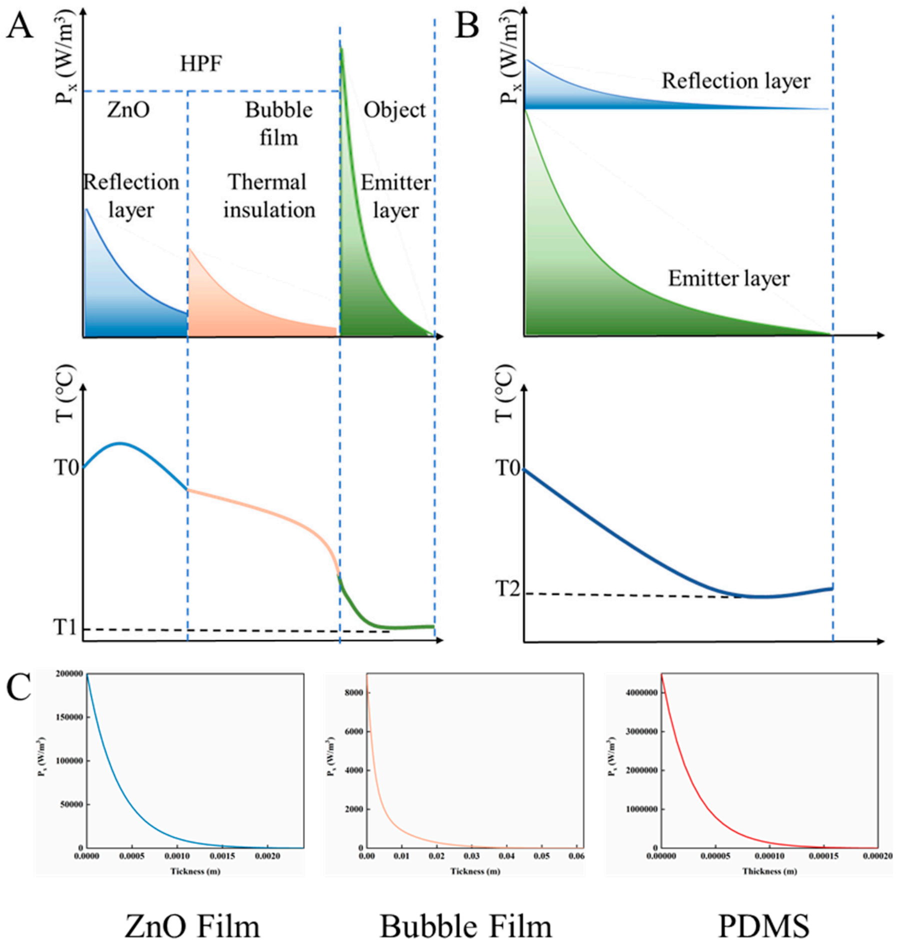

Figure S1 (A). Schematic of radiation distribution and cooling of each part of the HPF cooler. **(B).** Schematic of radiation distribution and cooling of each part of common radiation cooler**.** **(C)** Radiation capacity distribution of the three materials in the model calculation.

Supplementary Information 3: Model calculation: The effect of the sun's absorption depth on the cooling performance of the radiator

Based on Supplementary Information 1, the relationship between solar absorption depth and cooling performance is calculated by the model. The results show that, as the depth of absorption of the sun increases, the cooling performance becomes worse. When the sun's absorption depth is small, the energy absorbed by the sun can be dissipated during the heat exchange process with the environment, and will not significantly impact the cooling performance of the bottom layer.

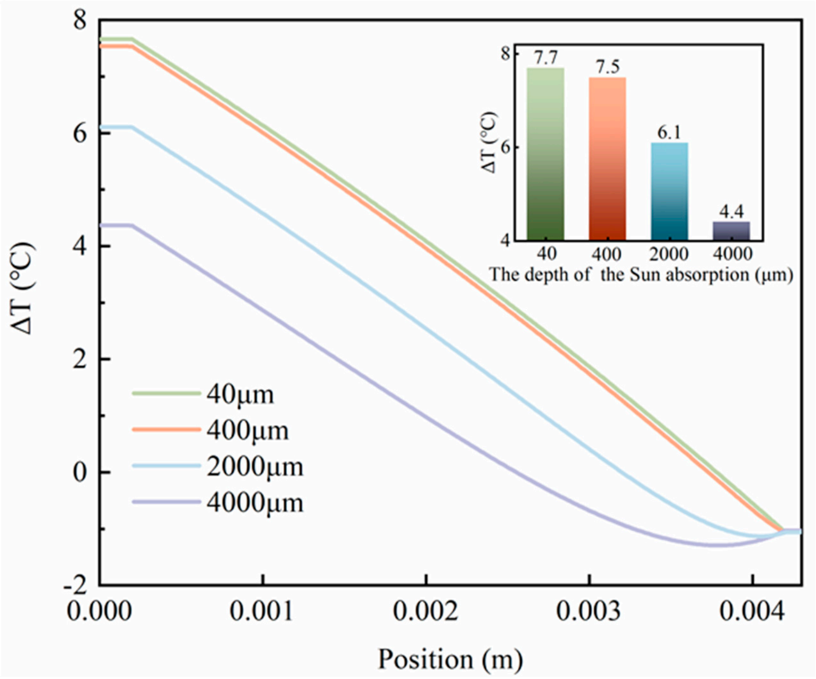

Figure S2. Solar absorption depth vs. temperature drop

Supplementary Information 4: Transmittance of PE, PP and POE film (all 25μm thickness) over MIR wavelength

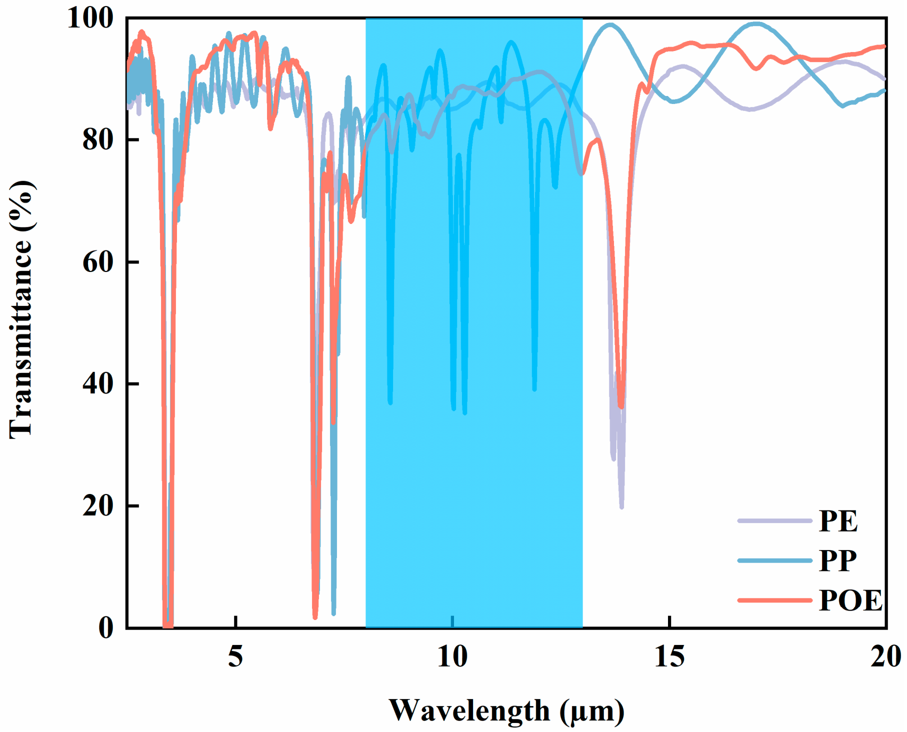

Figure S3. Transmittance of PE, PP and POE film

Supplementary Information 5: Evaluation of infrared transmission properties of different particles

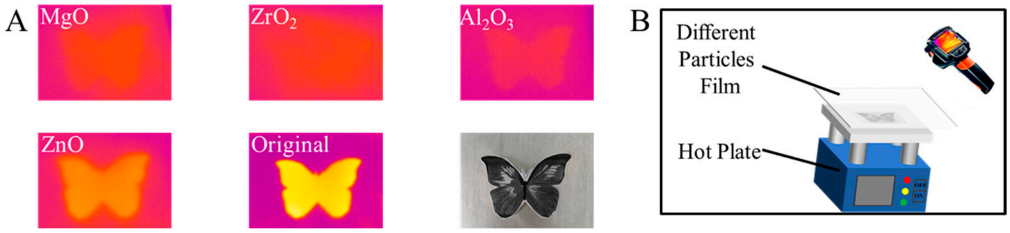

Figure S4 (A). Comparison of IR photos of materials (ZrO_2, MgO, Al_2O_3, ZnO) with the original PP film. They have the same particle size and thickness of the film layer. The IR photo of ZnO in nanoparticles is the clearest and the IR transmission is better. **(B).** Infrared photo shooting device: the butterfly pattern is placed on the heating table, the film is at a certain height from the heating table, and the position of them (including the film, the heating table and the infrared gun) is the same during each shoot.

Supplementary Information 6: Effect of particle size of ZnO on the normalized scattering cross section

Based on ZnO refractive index versus wavelength data [1], the normalized scattering cross section of individual ZnO spherical particles is calculated as the particle diameter varies from 0.01 to 10 μm in the wavelength range of 0.25-16 μm. As the particle size varies from 0.1 to 1 μm, the particles undergo strong Mie scattering, resulting in strong reflection in the solar band with less effect in the atmospheric window band.

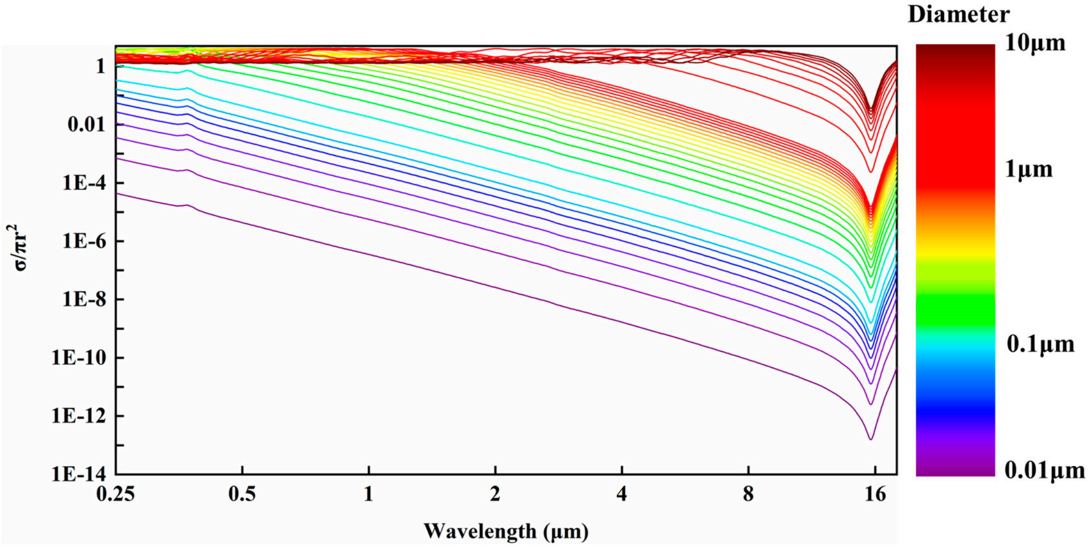

Figure S5. Particle size of ZnO vs. normalized scattering cross section

Supplementary Information 7: Particle choose

The ZnO used is not a regular sphere. The particle volumes were converted into equivalent spherical diameters by instrumental measurements. As shown in Figure S6, the equivalent spherical peak diameter of the particles used is about 500 nm.

The selected raw materials are cheap (the price shown in Table S1) and easy to get, which makes HPF easier scalable manufacturing.

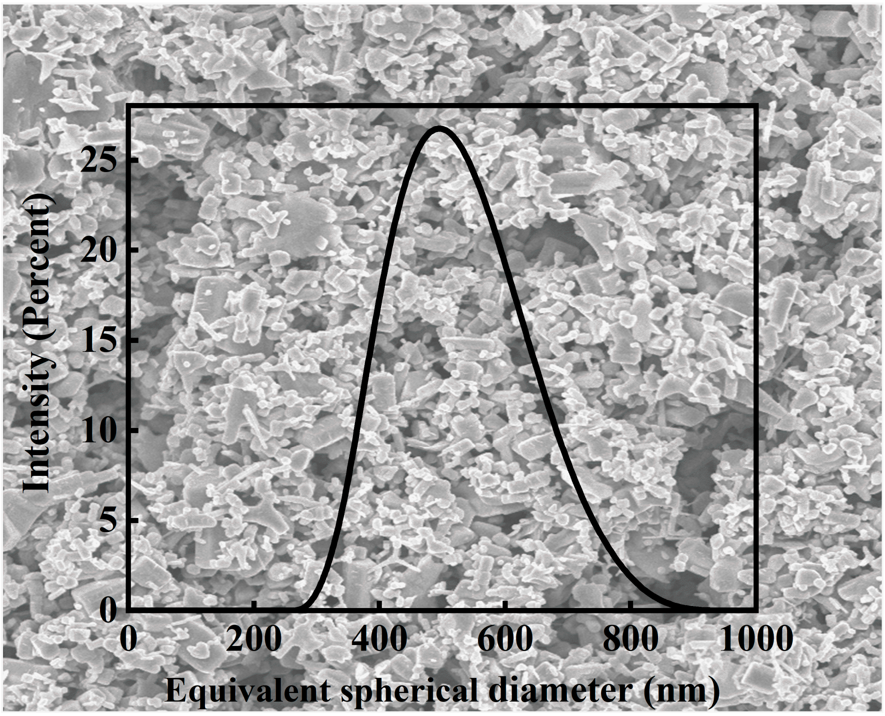

Figure S6. Particle size testing of ZnO

Supplementary Information 8: Various raw material price

Table S1. Various raw material price (data from www.alibaba.com)

Material	Price
POE	$3.1/kg
PP flim	$1.7/kg
PE flim	$2.5/kg
ZnO powder	$17.7/kg
Petroleum ether	$0.88/kg

Supplementary Information 9: Outdoor test experiment results of bubble films with different layers

Using a controlled variable experimental method, samples were set up with the same thickness of PDMS and ZnO film layers, in order to compare the cooling differences among 0 ~ 4 layers of bubble film (layer thickness is 2 mm).

The increase of bubble film layer reduces the heat exchange between the radiator and the environment, and decreases the transmittance of radiator's emission power. Considering that the bubble film itself also has a certain radiation performance, two layers of bubble film is the reasonable choice.

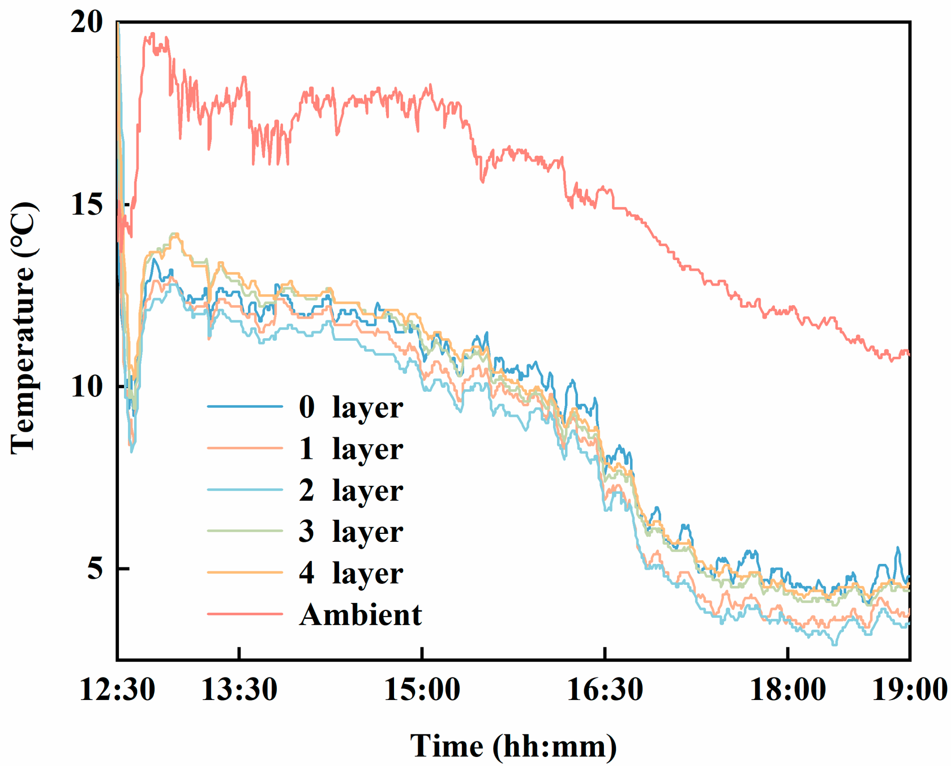

Figure S7. Effects of number of bubble film layers on cooling performance

Supplementary Information 10: Outdoor test experiment results of ZnO layer with different thickness

Using a controlled variable experimental method, samples with different thicknesses of ZnO layers were set up with the same thickness of PDMS and two layers of bubble films to compare the differences in cooling. The increases in the thickness of ZnO layer resulted in the decrease of both solar absorption and IR transmittance of the samples. The cooling performance increases with increasing thickness during daytime and decreases with increasing thickness during night time. However, there not obvious differences in the cooling performance. Due to the random outdoor conditions, it is difficult to deduce the regularity in outdoor tests at night.

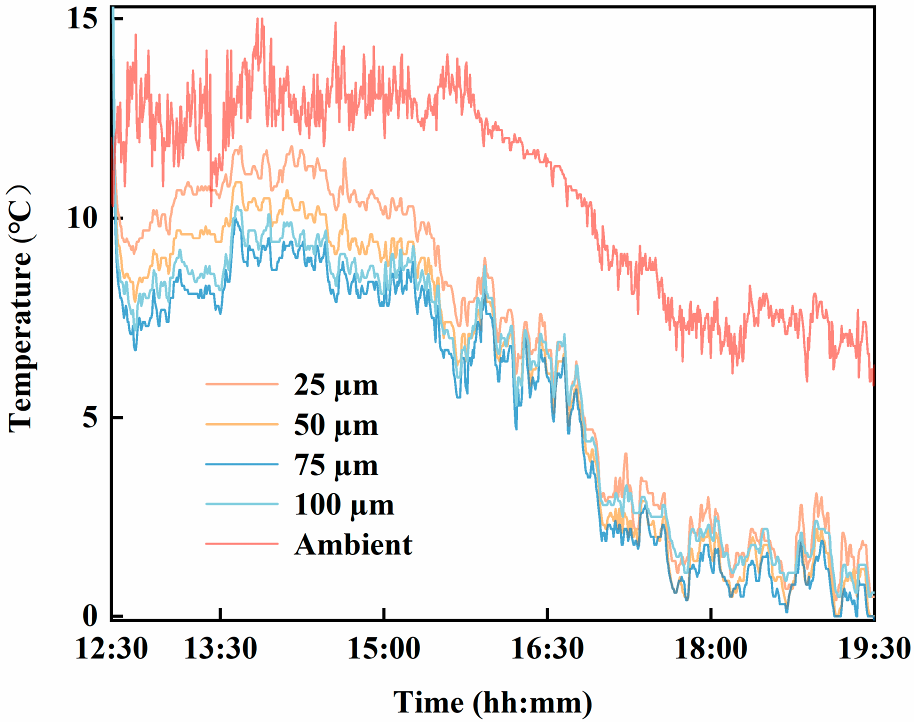

Figure S8. The cooling effects of ZnO layers with different thickness

Supplementary Information 11: Measurement of transmittance of HPF and emissivity of PDMS

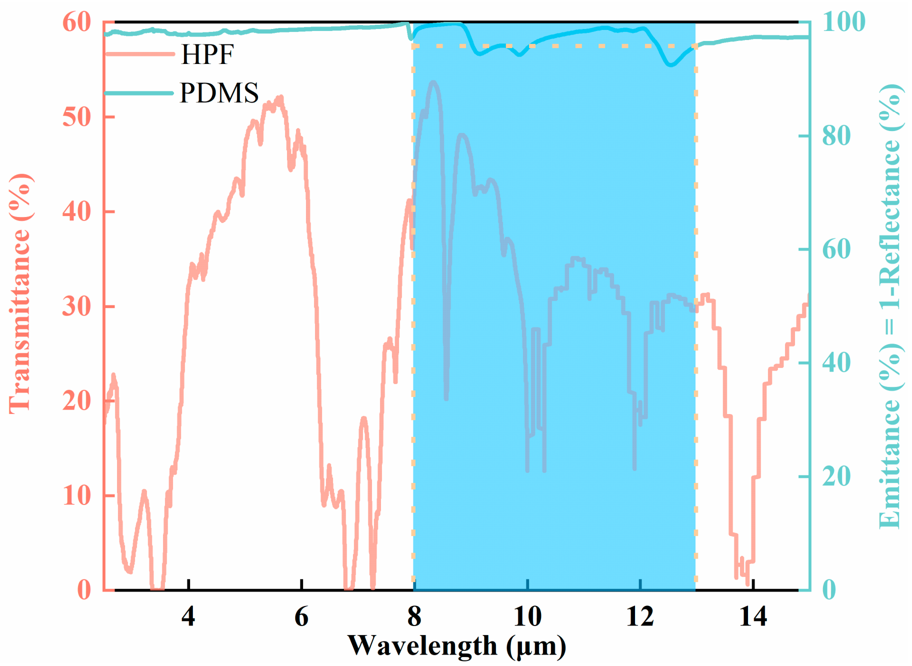

Figure S9. Transmittance of HPF (4.125μm thick) and emissivity of PDMS (200μm thick) over MIR wavelength

Supplementary Information 12: Comparison between different testing devices

Table S2. Comparison between different testing devices [2-4]

**Serial No.**	**Testing device**	**$\Delta T = T_{amb} - T_{sample}$**	**Description**
**1** (Limin Wu)	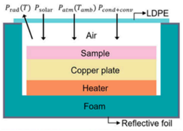	5.5℃	Foam box has relatively low thermal conductivity and can be well insulated from environmental heat transfer. High solar reflective aluminum foil reduces heat absorption from the box. PE film on the upper surface to reduce convective heat transfer between the environment and the sample.
**2** (Xiulin Ruan)	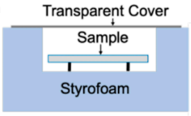	4.5℃	The purpose of the foam box setup is to prevent the chamber temperature from getting too high and affecting the testing of the cooling performance of the sample. However, due to the solar absorption of the aluminum foil and the chamber, the chamber temperature can be greater than the outside ambient temperature.<br>Compared with **1**, a gap between the sample and the foam box can reduce the heat transfer between the foam box and the sample.
**3** (Fuqiang Wang)	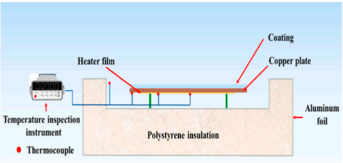	5.1℃	As the temperature of the foam box chamber is higher than the ambient temperature, covering PE film is more detrimental to the chamber heat dissipation.<br>The device removes the surface PE film on the basis of **2**. The sample is positioned slightly below the upper surface of the foam box, and this setup has a certain wind blocking effect. However, when the sun intensity is high, the inner surface of the foam box will still absorb heat and warm up.
**4**	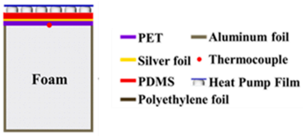	7.15℃	Our device is placed directly on the foam block with the sample facing the sun. Since the sample completely covers the upper surface of the foam block and the foam block is wrapped with aluminum foil around it, the foam block rarely comes into contact with the sunlight, so there is no heat absorption issue of the foam box. This setup is also in line with the actual application scenario of the samples. the test results are more informative.

Supplementary Information 13: Standard Deviation (*S*) of all-day outdoor test results

The smaller the standard variance value, the more stable the data. HPF is high thermal resistance, which can isolate the heat exchange between the environment and the radiant cooler. The HPF has the smallest standard variance in the all-day test results.

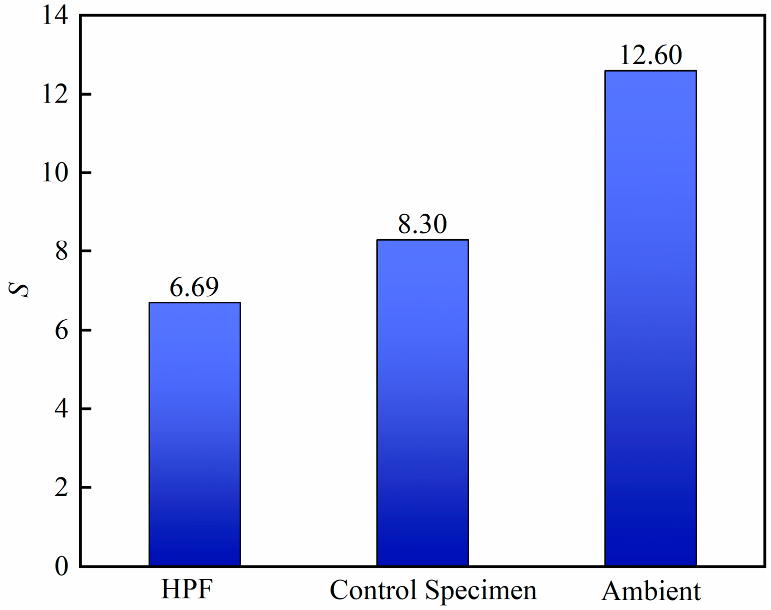

Figure S10. Standard variance of experimental results

Supplementary Information 14: Test of insulation effect of HPF

For a clearer view of the experimental results, tests were performed using ink-colored ice cubes. Three pieces of ice had the same mass. It can be seen form the Fig.S10, when placed on the heating table at 70°C, the ice without a cover melted completely within 11.5 minutes, while the ice covered by the HPF melted within 43 minutes. The latter's melted time is only 8 minutes shorter than that of the ice in the natural environment. It shows that HPF can effectively block the heat exchange between the heating table and the ice.

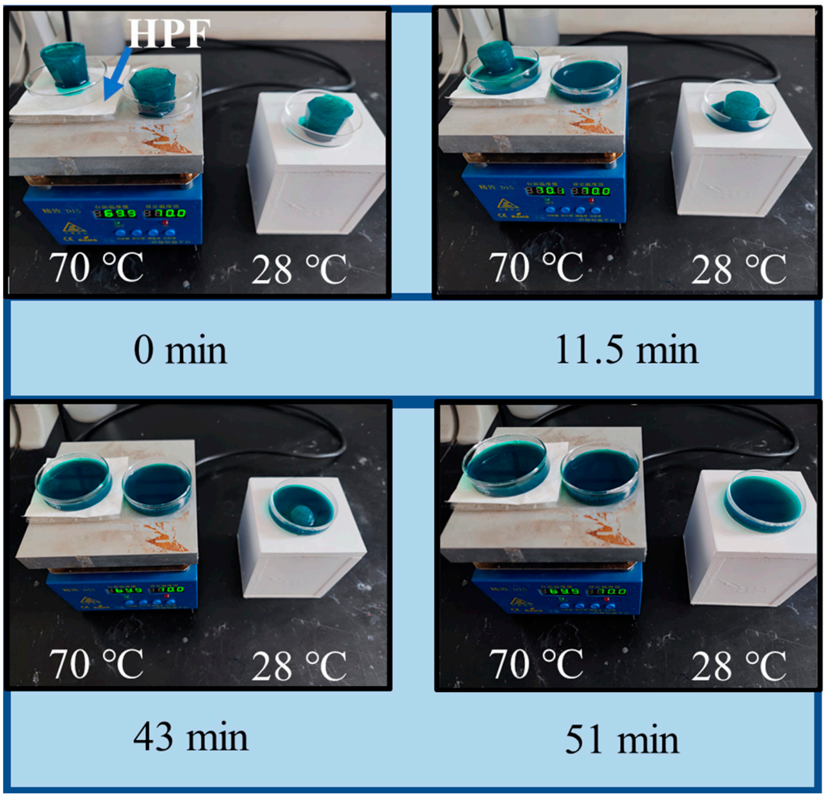

Figure S11. Test of insulation effect of HPF

Supplementary Information 15: Outdoor windshield experiments

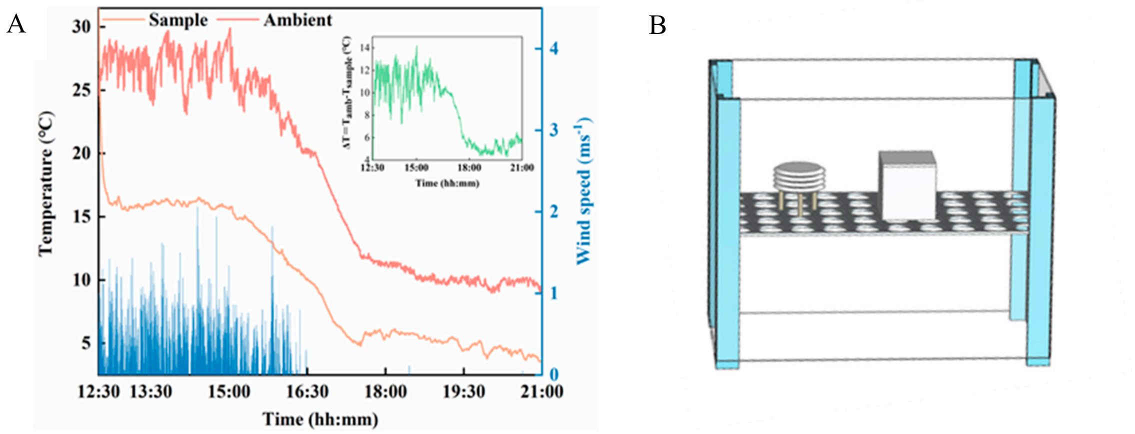

Figure S12. (A). Temperature measurement results of HPF in the windshield sub-environment. The left axis of the vertical coordinate is the temperature, the right axis is the wind speed. The average cooling was greater than 10°C during the day and greater than 5°C at night; **(B).** Windshield sub-environmental device: PE film was used to cover the surrounding area, and the sample and ambient thermometer were placed in the device. The wind speed was real-time monitored by an anemometer. Temperature fluctuation were monitored by thermocouples.

Supplementary Information 16: Outdoor cooling experiments on HPF-covered building materials

Actual cooling test results for building materials (including bitumen, cement and silicon carbide) are shown in Figure S13. The materials (silicon carbide≈200μm, bitumen≈500μm, cement≈2mm) are thick enough and have excellent infrared radiation capability. The cement was the thickest and responded the slowest to the environment. The most stable cooling activity is shown in the cement. It shows that HPF can achieve cooling of various objects with sufficient thickness.

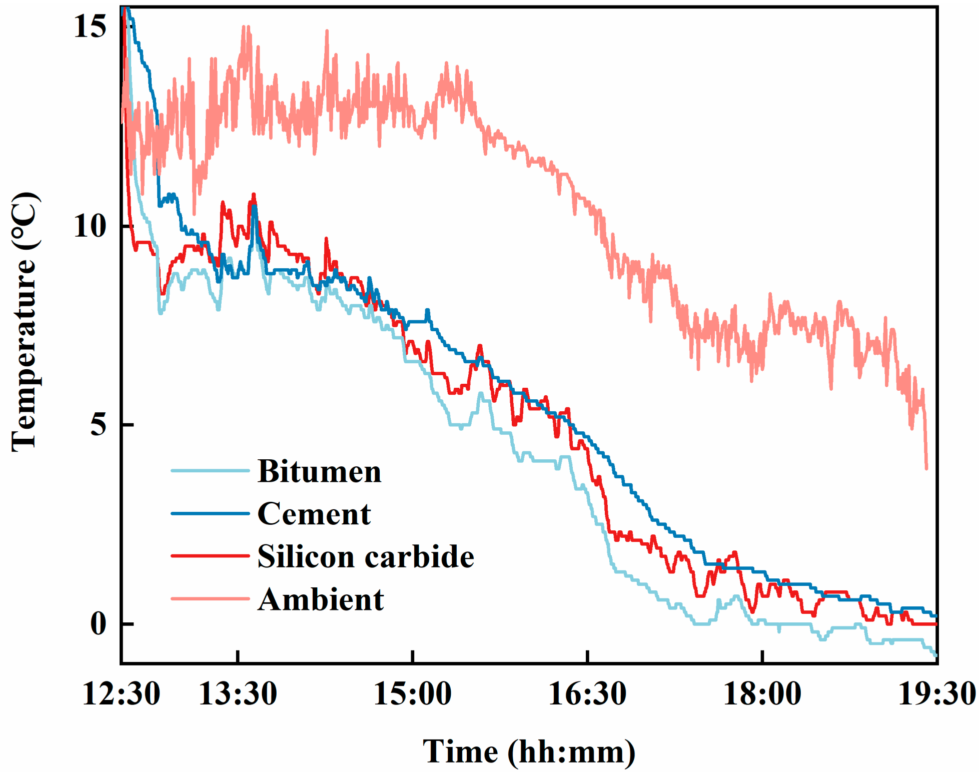

Figure S13. Cooling of HPF-covered building materials

Supplementary Information 17: The cooling performance of the material at different emitted and absorbed solar powers

Based on Supplementary Information 1, the effect of emitted power and solar absorption of the material on cooling was calculated. The higher the emitted power of the material and the lower the solar absorbed power, the better the cooling performance. When covered with HPF, the material can achieve cooling within the yellow dashed box by its own radiation capacity because the solar transmission is greatly blocked.

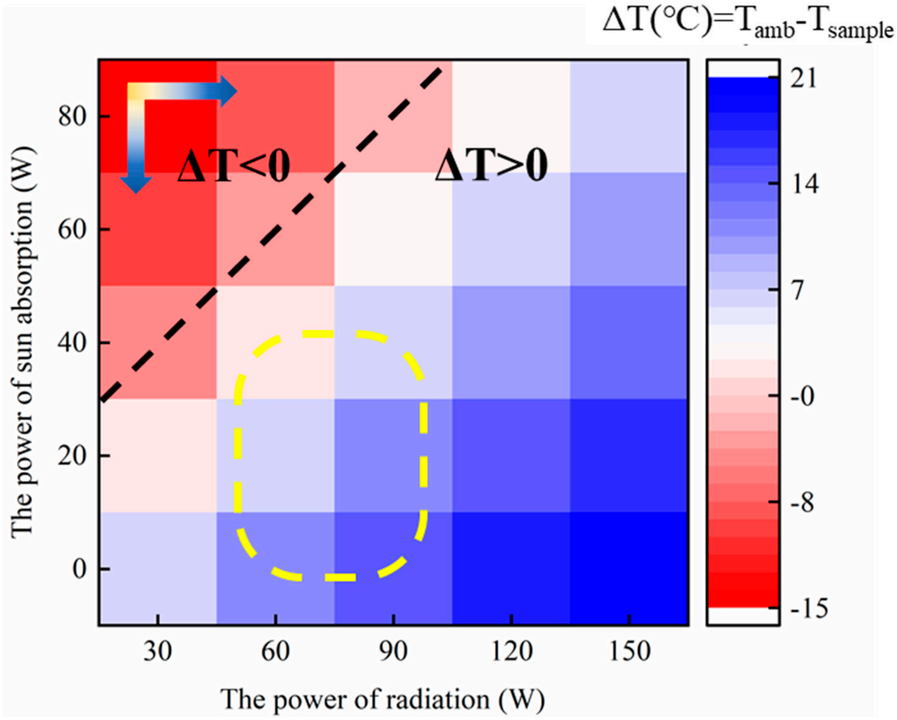

Figure S14. Hot spot diagram of the cooling performance of the material at different emitted and absorbed solar powers

Supplementary Information 18: High convection environment tests

When ZnO layer and bubble film were used, the average cooling of the sample was more than 3.5°C, which was better than that of pure PDMS (1.65°C). The number of bubble film layers has few effects on the cooling performance under high convection environment;

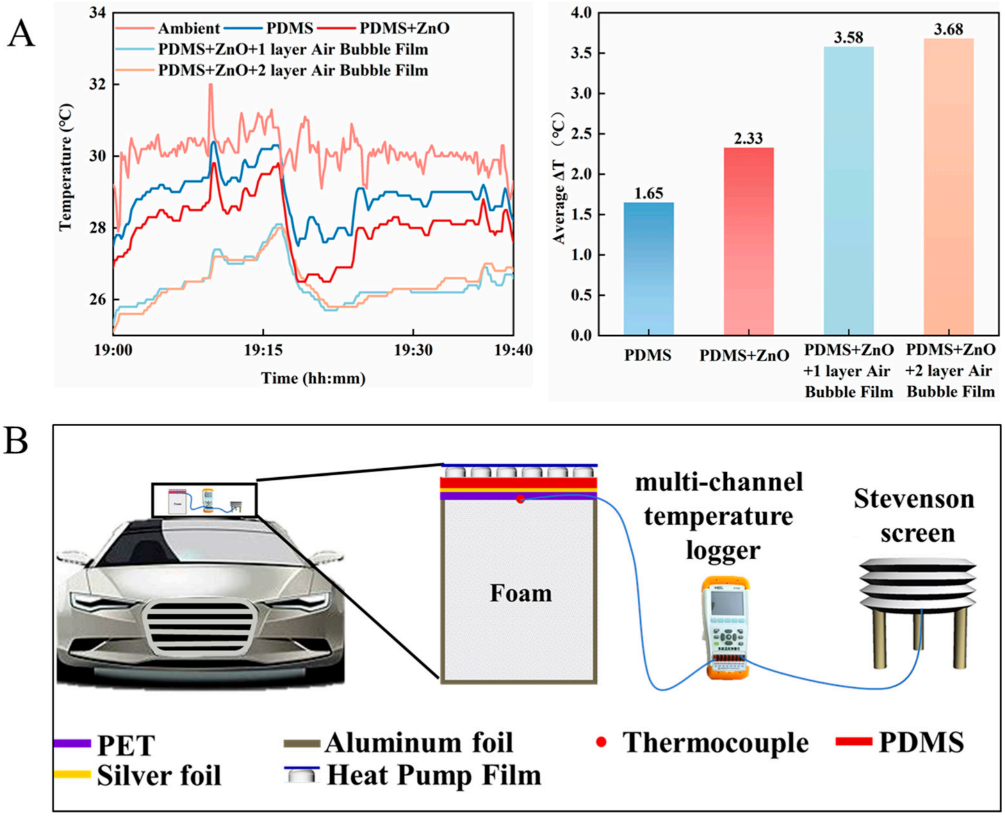

Figure S15 (A). Cooling test results under high convection conditions. **(B).** Test setup. The samples were placed on the roof of the vehicle. High speed of vehicle creates a high convection environment for samples. The cooling performance of the samples were tested on a driving car with 70km/h speed (the samples include PDMS, PDMS + ZnO layer, PDMS + ZnO layer + one-layer bubble film and PDMS + ZnO layer + two-layer bubble film).

Supplementary Information 19: Model calculation: Effects of surface convection heat transfer coefficients and number of bubble film layers

The cooling of the samples covered by bubble films with different layers. The surface heat transfer coefficient varies between 10~80 $W/m^2/k$. Figure S16(A) ~ (E) show the calculation results of 0, 1, 2, 3 and 4 layers of bubble films respectively. Figure S16(F) shows the location of each material. As the surface heat transfer coefficient increases, the cooling performance of the sample becomes worse and eventually stabilizes (surface heat transfer coefficient > 40 $W/m^2/k$).

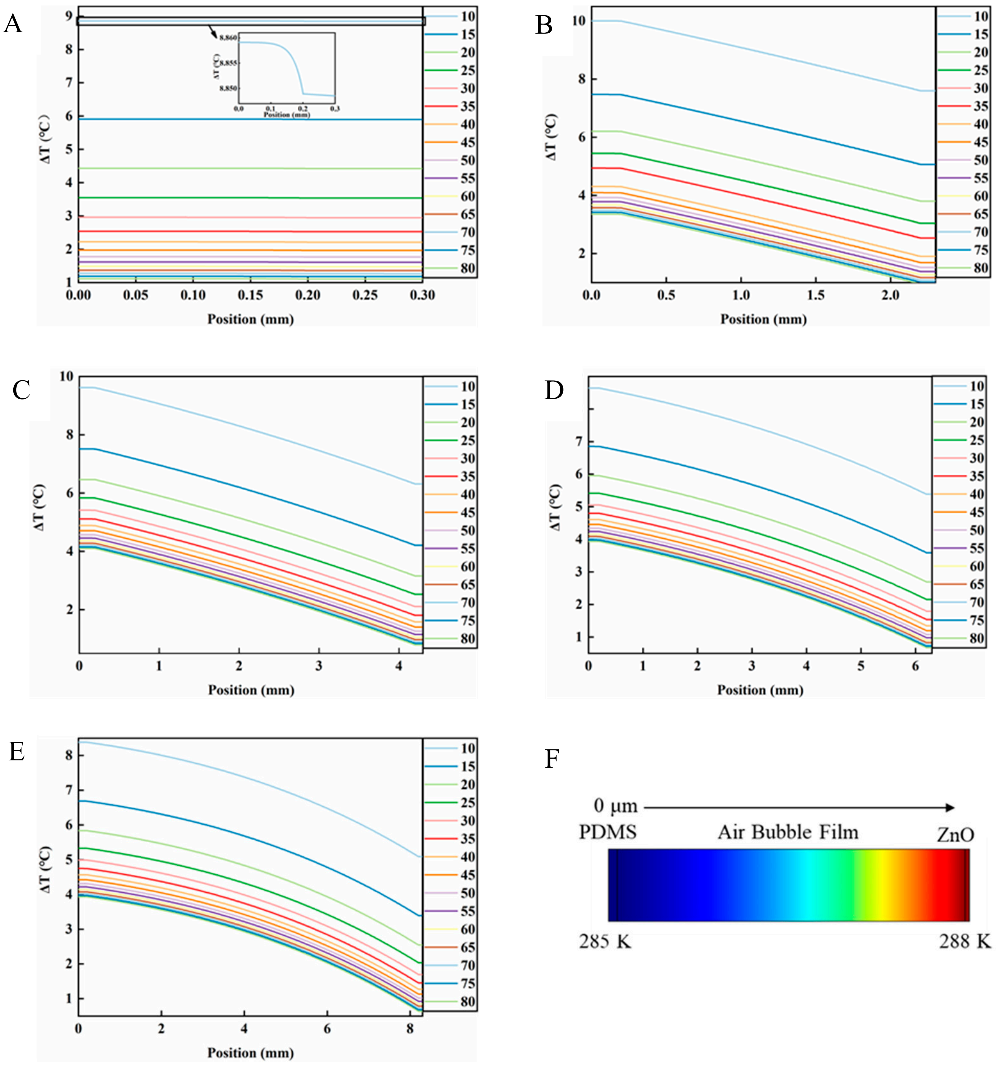

Figure S16. Effects of surface convection heat transfer coefficients and bubble film layers on cooling performance

Supplementary Information 20: Bending tests: HPF vs. PEA

As shown in Figure S17, HPF has excellent bending ability and recover ability compared with high foamed PEA.

A

B

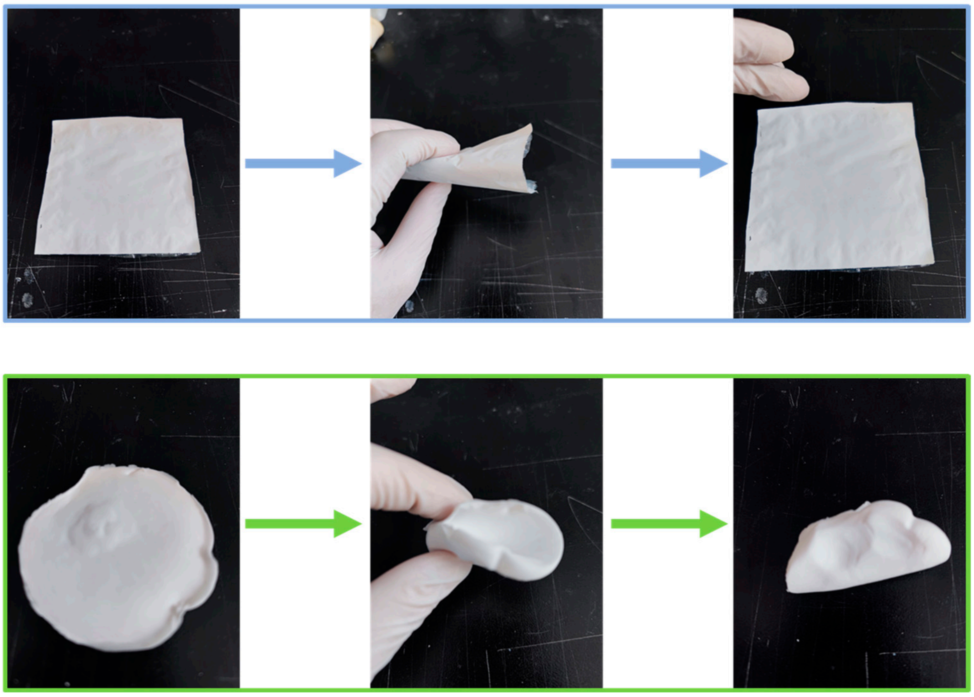

Figure S17. Bending tests

Supplemental Reference

[1] M. R. Querry. Optical constants, 1985, Contractor Report CRDC-CR-85034.

[2] Wang, T.; Wu, Y.; Shi, L.; Hu, X.; Chen, M.; Wu, L. A structural polymer for highly efficient all-day passive radiative cooling. Nat. Commun. 2021, 12, 20646. https://doi.org/10.1038/s41467-020-20646-7.

[3] Li, X.; Peoples, J.; Yao, P.; Ruan, X. Ultrawhite BaSO4 Paints and Films for Remarkable Daytime Subambient Radiative Cooling. ACS Appl. Mater. Interfaces 2021, 13, 21733-21739. https://doi.org/10.1021/acsami.1c02368.

[4] Cheng, Z.; Han, H.; Wang, F.; Yan, Y.; Shi, X.; Liang, H.; Zhang, X.; Shuai, Y. Efficient radiative cooling coating with biomimetic human skin wrinkle structure. Nano Energy 2021, 89, 106377. https://doi.org/10.1016/j.nanoen.2021.106377.
